# Techno-Economic Approach to Carbon Fibre Fabrics for Structural Strengthening: Life-Cycle Cost Analysis, Market Value, and Economic Viability

**DOI:** 10.3390/ma19101913

**Published:** 2026-05-07

**Authors:** Maciej Adam Dybizbański, Marceli Hązła, Alicja Krajewska, Katarzyna Rzeszut

**Affiliations:** 1 Faculty of Civil and Transport Engineering, Poznań University of Technology, 5 Marii Skłodowskiej-Curie Str, 60-965 Poznań, Poland; maciej.a.dybizbanski@gmail.com (M.A.D.); alicja.krajewska@put.poznan.pl (A.K.); 2 Department of International Economics, Poznan University of Economics and Business, Al. Niepodległości 10, 61-875 Poznań, Poland; marcelihazla@gmail.com

**Keywords:** life-cycle cost analysis (LCCA), carbon fibre-reinforced polymer (CFRP), structural strengthening, techno-economic analysis, infrastructure economics, durability, textile reinforced mortar (TRM), cost–benefit analysis

## Abstract

The escalating financial burden of deteriorating civil infrastructure worldwide necessitates a shift from conventional repair methods towards more durable and economically efficient long-term solutions. This paper presents a comprehensive techno-economic review of using carbon fibre-reinforced polymer (CFRP) fabrics for structural strengthening. Moving beyond a simple first-cost comparison, this review utilizes a life-cycle cost analysis (LCCA) framework to evaluate the total cost of ownership. The analysis deconstructs the complete cost profile, demonstrating that while CFRP systems have a high initial material cost, this is frequently offset by substantial savings in labour, equipment, and, critically, the indirect costs associated with reduced construction time and operational disruption. Furthermore, the inherent corrosion immunity of CFRP virtually eliminates future maintenance and repair expenditures, leading to a lower total life-cycle cost compared to traditional steel or concrete-based methods in a wide range of applications. Specifically, the conducted LCCA case study demonstrates that the CFRP alternative can reduce total life-cycle costs by nearly 25% relative to conventional steel sheet bonding, overwhelmingly driven by minimized operational downtime and related indirect costs. The value proposition is shown to be context-dependent, driven by minimizing user delay costs in bridges, mitigating catastrophic risk in seismic retrofitting, preserving cultural value in heritage structures, and maximizing revenue uptime in industrial facilities. The review also examines market dynamics, including the roles of standardization and government policy in driving adoption, and explores future trends such as inorganic matrix composites (TRM/FRCM), integrated structural health monitoring (SHM), and the push towards a circular economy. The findings conclude that a holistic, life-cycle-based economic assessment establishes CFRP strengthening as a cornerstone technology for the sustainable and resilient management of modern civil infrastructure.

## 1. Introduction

Civil infrastructure is the foundational pillar of the global economy, enabling trade, commerce, and societal mobility. This critical asset, however, is facing a pervasive and systemic challenge: accelerated deterioration. Across the developed world, a significant portion of bridges, transportation networks, and public buildings, largely constructed in the post-war boom of the mid-20th century, are now approaching or have already exceeded their original design life. The failure to adequately invest in their upkeep has created a substantial financial burden, with economic consequences that extend far beyond the direct costs of repair.

For instance, the American Society of Civil Engineers (ASCE) estimates that the United States faces an infrastructure investment gap of $2.59 trillion over ten years [1]. If this deficit is not addressed, the cumulative cost to the U.S. economy could reach $10 trillion in lost GDP by 2039, with the average American household losing $3300 annually due to infrastructure deficiencies [1]. This challenge is not unique to North America. European nations face similar predicaments, with countries like Germany reporting a significant maintenance backlog for thousands of its bridges on the critical Trans-European Transport Network (TEN-T) [2].

The financial burden manifests through two primary channels: direct and indirect costs. Direct costs are associated with the escalating expense of deferred maintenance. In principle, minor, timely interventions are significantly more cost-effective than major, reactive repairs. Studies have shown that for bridge maintenance, every $1 not spent on preventative care can lead to $4 to $5 in required future capital repairs, as minor issues like water ingress cascade into major problems like rebar corrosion and concrete spalling [3]. Perhaps even higher are the indirect costs, which are borne by society and the wider economy, as deteriorating infrastructure leads to reduced efficiency and reliability. For example, weight-limited bridges force commercial vehicles onto longer, more costly detour routes, disrupting supply chains and increasing transportation expenses [4]. Chronic traffic congestion resulting from poor road conditions and constant repair work leads to lost productivity, increased fuel consumption, and higher vehicle operating costs [5].

In response to those challenges, engineers have historically relied on a set of established, conventional strengthening techniques. These methods, primarily including steel plate bonding, section enlargement via concrete jacketing or shotcreting, and external post-tensioning, can effectively restore or enhance the load-carrying capacity of structural elements. However, a critical examination of their economic profile reveals that they often introduce significant long-term liabilities, high indirect costs, and logistical complexities that diminish their overall value proposition in a modern asset management context [6]:

- Steel plate bonding, while structurally effective initially, suffers from the susceptibility of steel to corrosion. The harsh interface between the steel plate, adhesive, and the existing concrete creates an environment ripe for moisture ingress and electrochemical processes, leading to corrosion that can cause delamination and a sudden, premature loss of the strengthening effect [7].
- Section enlargement has a significant drawback of the addition of significant dead load to the structure. This added mass not only increases the seismic forces the structure must resist but may also overstress other elements, such as foundations, which may then require their own costly strengthening interventions [8].
- External post-tensioning demands specialized equipment, highly skilled labour, and sophisticated design, making its initial capital cost prohibitive for many applications. More importantly, the long-term economic viability is challenged by the critical need for maintenance and inspection of its components [9].

The search for a more economically sustainable and structurally efficient alternative to conventional repair methods has led to the widespread adoption of advanced composite materials, most notably carbon fibre-reinforced polymers (FRPs), consisting of high-strength structural fibres embedded within a polymer matrix, offering an unparalleled combination of mechanical properties and durability [10].

The foremost advantage of CFRP is its inherent immunity to electrochemical corrosion, which is a big problem in traditional repair methods [11]. Furthermore, CFRP composites possess an exceptionally high strength-to-weight ratio, reducing the need for heavy lifting equipment, scaffolding, and large labour crews [10], therefore generating enormous savings in indirect costs [12]. While the initial purchase price of carbon fibre materials is higher than that of steel or concrete, a comprehensive life-cycle cost analysis (LCCA) often reveals CFRP to be the more economically advantageous option. When the total cost of ownership—including reduced installation time, minimized indirect costs from disruption, and the near-elimination of future maintenance expenditures—is considered, the lifetime cost of a CFRP solution is frequently lower than that of its traditional counterparts [13].

The field of CFRPs for structural applications is mature, and its technical aspects have been the subject of many comprehensive review articles over the past three decades [10,14]. Subsequent research has provided deep insights into specific challenges such as long-term durability, fire performance, and bond behaviour [11]. However, the majority of this body of literature maintains a primary focus on technical performance and engineering mechanics. While the economic benefits are often mentioned qualitatively, a holistic review that systematically integrates these technical characteristics with a rigorous economic framework—specifically from a life-cycle cost and value perspective—is less developed. Asset owners, infrastructure managers, and policymakers often make investment decisions based on long-term economic viability, yet there is a gap in the literature that would bridge the areas of engineering and finance [15].

Therefore, the primary objective of this paper is to provide a comprehensive techno-economic review of carbon fibre fabrics used for structural strengthening. To achieve this, the paper will pursue the following specific objectives:

1. To deconstruct the complete cost profile of CFRP strengthening systems;
2. To establish life-cycle cost analysis (LCCA) and cost–benefit analysis (CBA) as the definitive frameworks for evaluating the economic feasibility of CFRP;
3. To analyze the key market dynamics;
4. To evaluate the economic outlook and potential impact of emerging trends.

By synthesizing technical performance with economic rationale, this review aims to equip a diverse audience—including practicing engineers, asset managers, researchers, and public-sector decision-makers—with the comprehensive knowledge required to make informed, value-driven investment decisions for the sustainable and resilient rehabilitation of global civil infrastructure.

## 2. Deconstruction of Costs

### 2.1. Material Costs

The material cost represents the single largest component of the initial capital expenditure in most CFRP strengthening projects. Unlike traditional materials like steel and concrete, whose prices are relatively low and well-established, the cost of advanced composites is significantly higher and driven by complex manufacturing processes and volatile commodity markets.

- The high cost of carbon fibre itself is the primary economic barrier to its wider adoption. This price is a direct consequence of its highly energy-intensive and technologically complex manufacturing process [16]. As of the early 2020s, prices for standard, industrial-grade carbon fibre typically ranged from $20 to $40 per kilogram, although this is subject to significant market volatility tied to the price of oil and electricity [17]. High-modulus or high-strength fibres, which require even higher processing temperatures and greater precision, can be several times more expensive.
- The Polymer Matrix System is used to impregnate the dry carbon fabric and bond it to the substrate. For structural strengthening applications, high-performance, two-part epoxy resin systems are the industry standard. This is due to their excellent adhesive properties, high mechanical strength, low creep, and proven durability in harsh environments [18]. The complete system supplied by a manufacturer typically includes several components, each with an associated cost:
  ○ A low-viscosity epoxy primer to penetrate the prepared concrete substrate and ensure a strong bond.
  ○ The primary epoxy saturant, which is mixed and used to impregnate the carbon fabric during wet layup.
  ○ Sometimes, a putty-like epoxy is also required for filling small surface voids to create a smooth substrate.

Beyond the resin system itself, a project’s bill of materials must also include ancillary consumables such as specialized application rollers, brushes, mixing equipment, and personal protective equipment (PPE). While individually minor, these items collectively add to the total material expenditure [19].

To contextualize the economic baseline, Table 1 provides a comparative overview of typical unit material costs for the three primary structural strengthening systems. While raw material prices fluctuate based on global supply chains and energy markets, the table clearly illustrates the substantial initial material premium associated with CFRP compared to conventional alternatives.

### 2.2. Labour and Equipment Costs

While the high material costs present a significant upfront investment, a substantial portion of this expenditure is frequently offset by considerable savings in labour and equipment, as application of CFRP fabrics is a skilled but not necessarily strength-intensive task [10]. A typical installation crew consists of a small team of 2–4 certified technicians responsible for surface preparation, mixing the epoxy resin, and applying the fabric using simple hand tools like rollers and squeegees [18]. Rolls of carbon fibre fabric are light enough to be carried and manipulated by hand, eliminating the need for mechanical assistance for material handling. As a result, case studies performing direct comparisons have documented that CFRP installation can lead to reductions in total person-hours of up to 50–70% compared to equivalent repairs using conventional methods [20].

The disparity in equipment requirements is equally significant. A typical CFRP strengthening project requires relatively light and inexpensive equipment: handheld grinders for surface preparation, power mixers for the resin, and standard access equipment like mobile scaffolding or aerial man-lifts. This minimalist approach contrasts sharply with the heavy-duty machinery essential for traditional methods, requiring heavy lifting or concrete mixing, among others [21].

It is important to note that while the overall labour demand is lower, the installation of CFRP is a specialized skill. Reputable industry standards and guides mandate that applicators be properly trained and certified by the material manufacturer to ensure quality control and proper performance of the system [18]. This means the hourly wage for a certified FRP technician may be higher than that of a general construction labourer. Wages for skilled technicians can be 5–15% higher than the industry average [22], and on highly specialized projects, workers may receive pay increases of 25–30% compared with previous wages due to extremely high demand [23]. However, this premium for skilled labour is almost always overwhelmingly compensated for by the dramatic reduction in the total number of hours and personnel required to complete the project.

### 2.3. Indirect Costs

While direct costs associated with materials and labour are straightforward to quantify, the economic competitiveness of CFRP strengthening is most profoundly revealed through an analysis of indirect costs. These costs, often overlooked in simplistic bid comparisons based solely on initial outlay, represent the real-world financial impact of a construction project on the asset’s owner, its users, and the surrounding economy [24].

For public infrastructure, particularly in transportation networks, these are most accurately termed “Work Zone Road User Costs” (WZRUC). These costs do not appear on a contractor’s invoice but represent a very real economic loss to society. Authoritative bodies like the U.S. Federal Highway Administration (FHWA) categorize them into three main components:

1. The value of time lost by motorists, both private and commercial, due to traffic congestion, detours, and reduced speeds. For commercial freight, this translates into direct productivity losses and supply chain disruptions.
2. The increased expense from fuel consumption, tyre wear, and vehicle depreciation caused by stop-and-go driving conditions or travelling longer distances via detours.
3. The societal cost associated with the statistically higher probability of traffic accidents occurring in and around work zones due to lane shifts, merging, and other non-standard conditions.

For major transportation arteries, these user costs can often reach tens or even hundreds of thousands of dollars per day. The key advantage of CFRP is speed. A repair that might take several weeks with concrete jacketing, requiring prolonged lane closures, can often be completed in a matter of days or overnight with CFRP wraps. Case studies analyzing bridge repairs have demonstrated that these multi-week time savings can translate into millions of dollars in avoided user delay costs.

The magnitude of WZRUC can often exceed the direct construction costs, particularly on high-traffic corridors. For example, analyses conducted by the Federal Highway Administration (FHWA) indicate that user delay costs on major highways may reach tens of thousands to over $100,000 per day, depending on traffic volume and detour length. In heavily trafficked urban corridors, total user delay costs during bridge rehabilitation projects have been reported to exceed several million dollars over the project duration [25].

This principle extends beyond public infrastructure to private and commercial facilities, where indirect costs manifest as direct revenue loss for the owner. For revenue-generating assets, every hour of downtime has a quantifiable price tag, be it in industrial facilities, commercial buildings or port and marine structures [26]. In these contexts, the speed of a CFRP repair is not just a convenience; it is a direct driver of return on investment (ROI). Therefore, the mitigation of indirect costs is arguably the most compelling component of CFRP’s economic value proposition. It shifts the decision-making paradigm from a narrow focus on the “cost of the repair” to a holistic view of the “cost of the structure being out of service.” This effect is particularly pronounced in urban bridge repairs, where the economic benefit of avoided user delay costs can justify the entire project.

### 2.4. Engineering, Design, and Quality Assurance Costs

Beyond the direct “hard costs” of materials and on-site execution, a complete economic profile must also account for the “soft costs” associated with engineering design, inspection, and quality assurance (QA). For CFRP strengthening projects, these costs can initially be higher than for conventional methods due to the specialized knowledge required for both design and verification.

The design of externally bonded CFRP systems, while well-codified in established international guides like the ACI 440.2R-17 [18], differs significantly from traditional steel and concrete design. Engineers must account for the anisotropic properties of the composite material and design for unique, and often brittle, failure modes such as laminate debonding or delamination, which are not present in conventional monolithic structures [27]. This requires a level of specialized expertise that may not be available in-house at all engineering firms. Consequently, design costs can be elevated due to higher billing rates for specialist engineers or the need to subcontract the design to a niche consulting firm. However, as the technology has matured, this cost premium has been steadily decreasing due to the development of dedicated design software and a growing pool of experienced engineers [17].

Arguably more critical from a cost and performance perspective is the investment in a robust quality assurance and quality control (QA/QC) programme. The final performance of an installed CFRP system is exceptionally sensitive to the quality of the application, including substrate preparation, resin mixing, and fabric saturation. A failure in any of these steps can compromise the entire system. Therefore, a rigorous QA/QC plan is an essential project cost. This typically includes continuous visual inspection, post-cure inspection and adhesion testing [28]:

For highly critical applications, more advanced and costly non-destructive testing (NDT) methods may be specified, such as infrared thermography, which can identify voids and delaminations by detecting thermal gradients, or ultrasonic testing [29]. Ultimately, these engineering and quality assurance expenditures should be viewed not as mere costs, but as an essential investment in ensuring the project’s success and achieving its full design potential.

## 3. Life-Cycle Cost Analysis (LCCA) of CFRP Strengthening

### 3.1. Principles of LCCA in Construction

**Figure 1:**
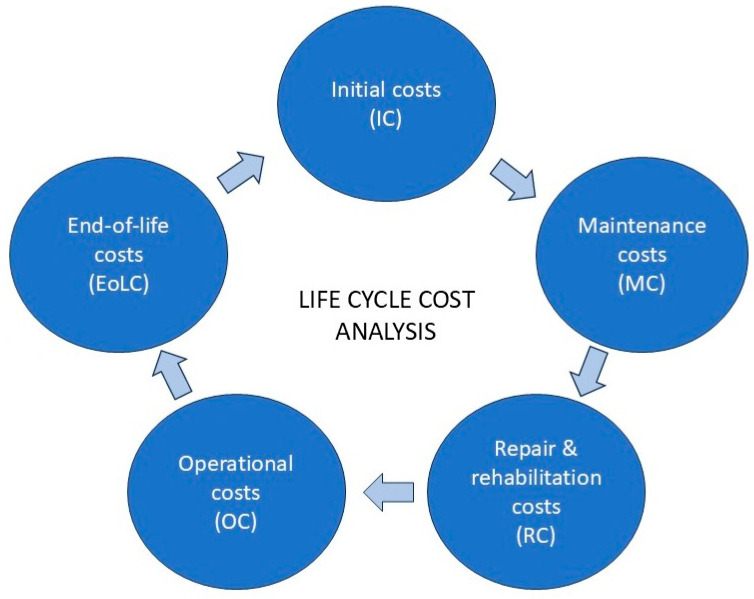
LCCA framework. Source: [31].

To move beyond the limitations of a simple initial-cost comparison, a more rigorous and comprehensive economic methodology is required. Life-cycle cost analysis (LCCA) provides this framework, serving as the standard tool for evaluating the long-term economic viability of competing project alternatives with different profiles of initial and future expenditures (Figure 1). Formally defined by ISO 15686-5, LCCA is a technique used to estimate the total cost of ownership of a constructed asset over its entire operational life, or a specified period of analysis [30]. Its primary objective is to provide a transparent and objective basis for decision-making, ensuring that choices are based on the best long-term value rather than the lowest initial price [31].

The fundamental principle underpinning LCCA is the time value of money. This economic concept recognizes that a sum of money today is worth more than the same sum in the future due to its potential earning capacity (opportunity cost) and the effects of inflation. To account for this, all future costs incurred throughout the analysis period are converted to their equivalent present-day value using a mathematical process called discounting. The rate used for this conversion is the discount rate, a critical parameter representing the investor’s minimum acceptable rate of return or the societal cost of capital. Usually for the discount rate in construction projects, a national treasury bond discount rate (most often for the 10-year ones) is used [32].

The primary metric derived from an LCCA is the Net Present Value (NPV) of the total life-cycle cost. This single figure represents the sum of all discounted costs over the analysis period. The decision rule is straightforward: when comparing mutually exclusive alternatives for a project, the option with the lowest total life-cycle cost (lowest NPV) is the most economically favourable choice.

The total life-cycle cost (LCC) can be expressed conceptually by the following formula, where all future costs are discounted to their present value:

(1) $$LCC=IC+NPV*\left(MC+RC+OC+EoLC\right)-NPV\left(VR\right),$$

where

ICs (Initial Costs)—The total upfront cost of the project (materials, labour, equipment, design, and QA/QC).

MCs (Maintenance Costs)—The sum of all planned, routine maintenance activities required to keep the asset at its functional level (e.g., routine inspections).

RCs (Repair and Rehabilitation Costs)—The costs of any future major repairs or replacements anticipated during the analysis period (e.g., replacing a protective coating on steel plates).

OCs (Operational Costs)—The costs associated with the asset’s operation, such as indirect user costs (e.g., from traffic delays during future maintenance activities) discussed earlier.

EoLCs (End-of-Life Costs)—The costs associated with decommissioning, demolition, and disposal of the asset or strengthening system.

RV (Residual Value)—The value of the asset at the end of the analysis period, which is treated as a credit.

To ensure a valid comparison, a consistent analysis period (e.g., 50, 75, or 100 years) must be established and applied to all alternatives [31]. By translating all future expenditures and benefits into a single, present-day value, LCCA provides a rational basis for decision-making. The following sections will apply this framework to demonstrate how the unique properties of CFRP composites influence each component of the life-cycle cost equation, ultimately building the case for their long-term economic advantage. For low-probability, high-consequence events, this framework is adapted into a cost–benefit analysis (CBA) to evaluate the immense value of avoided catastrophic losses, for instance, in the seismic retrofitting of columns.

### 3.2. Initial Investment Analysis (CI)

The initial investment, also known as capital expenditure (CAPEX), represents the first term (IC) in the life-cycle cost analysis equation. This includes the combined costs of materials (fabrics, resins), labour (including specialty technicians), equipment, engineering design, and rigorous quality assurance. Crucially, for a methodologically sound LCCA, the CI must also incorporate the significant indirect costs incurred during the construction period, such as road user costs or owner revenue loss [31]. When comparing project alternatives, it is a well-documented observation that the initial costs (ICs) for a CFRP strengthening solution are often higher (even by 20–50%) than that for conventional methods like steel plate bonding or concrete jacketing [33]. This upfront cost premium, driven primarily by the high price of the advanced composite materials themselves, is frequently the primary reason for decision-makers to hesitate in adopting the technology.

However, viewing this initial cost in isolation is fundamentally misleading, as the composition of the cost differs dramatically between the alternatives. For a conventional project, a large percentage of the IC is composed of labour, heavy equipment rental, and, most significantly, the extensive indirect costs resulting from prolonged project durations. For a CFRP project, the cost profile is inverted: a higher percentage is allocated to the advanced materials, while the costs for labour, equipment, and indirect impacts are substantially lower due to the speed and efficiency of installation.

The conventional “lowest first-cost” procurement model is ill-suited to evaluate such trade-offs. By focusing exclusively on minimizing the initial outlay, it systematically favours methods that may be cheaper upfront but generate significant, un-costed disruptions and lock in higher future maintenance liabilities [34]. A proper LCCA framework corrects this flaw by first establishing a comprehensive IC that includes all direct and indirect costs, thereby providing a true and fair baseline for comparison.

Furthermore, the LCCA must explicitly account for the operational environment, particularly in building applications where strict fire resistance is critical. Conventional epoxy-based CFRP systems suffer from severe bond deterioration at moderate temperatures (100–200 °C) [35]. Consequently, to achieve fire ratings comparable to concrete, they require supplementary passive fire protection (PFP), such as intumescent coatings or 20–50 mm thick insulation boards [35]. The mandatory installation of PFP materially alters the economic profile by increasing the initial costs through additional materials and specialized labour, while simultaneously introducing new long-term maintenance costs. In such fire-critical scenarios, the overarching economic advantage of CFRP is significantly narrowed, making inorganic matrix composites (e.g., TRM/FRCM) or traditional concrete jacketing potentially more cost-effective alternatives [36].

### 3.3. Maintenance, Repair, and Operational Cost Analysis (MC, RC, OC)

The primary economic justification for the higher initial investment in CFRP systems lies in the reduction in future expenditures. An analysis of the maintenance, repair, and operational cost streams within the LCCA framework reveals the profound long-term financial advantage of CFRP’s inherent durability.

The most significant driver of this long-term saving is the immunity of CFRP composites to electrochemical corrosion. Unlike steel, which begins a relentless battle against oxidation from the moment it is installed, CFRP materials are chemically inert and do not degrade in the presence of moisture, chlorides, or other aggressive agents common in civil infrastructure environments [11]. This inherent material stability virtually eliminates the single largest driver of future maintenance and repair costs that plagues conventional steel strengthening systems. In stark contrast, a structure strengthened with bonded steel plates requires a costly and perpetual maintenance program. Depending on the environmental severity, a complete sandblasting and repainting of the steel plates—a major capital project in itself—is typically required every 20–25 years, with costs that can run into the hundreds of thousands or millions of dollars for a single bridge [37].

When translated into the LCCA framework, this disparity is striking. For a CFRP alternative, the maintenance (MCs) and repair costs (RCs) are minimal, often limited to the low cost of periodic visual inspections. For the steel alternative, these terms are substantial, representing a recurring, high-cost liability that must be discounted and added to its total life-cycle cost. Published LCCA case studies comparing strengthening alternatives for bridges have demonstrated this effect quantitatively. While the steel plate option often has a lower initial cost, its LCC typically surpasses that of the CFRP option, often within a 15–20 year timeframe, due to the heavily discounted cost of just one or two major repainting cycles [12]. Furthermore, these future maintenance activities have an operational cost component (CO). Each time a conventional repair requires invasive maintenance (e.g., repainting steel plates), a new work zone must be established. This triggers a new wave of indirect costs from traffic disruption and user delays.

### 3.4. End-of-Life (EoL) and Residual Value Analysis (EoLC, VR)

A complete and forward-looking life-cycle cost analysis must extend to the very end of the asset’s service life, considering the costs of decommissioning and disposal (EoLCs), as well as any potential scrap or resale income (residual value, VR) [30]. This EoL phase, increasingly scrutinized through the lens of sustainability and the circular economy, reveals a significant economic disparity between conventional materials and current CFRP composites. At present, it represents one of the few areas in the LCCA where traditional materials hold a distinct advantage.

For conventional strengthening systems using steel, the end-of-life scenario is economically favourable, as steel is one of the most recycled materials globally, with a mature, efficient, and profitable collection and reprocessing industry. Recycling rates for structural steel consistently exceed 85–90% [38]. This means that at the end of a structure’s life, the removed steel plates have a positive market value as scrap. In LCCA terms, the revenue generated from selling the scrap steel (VR) can substantially offset or even exceed the labour and equipment costs of removal (CEoL), resulting in a net negative cost (i.e., a net gain) for this phase of the life-cycle.

The end-of-life outlook for CFRP composites is currently far more challenging. Due to their composite nature, separating the components for high-value recycling is difficult and energy-intensive. Consequently, the most common disposal route for CFRP waste from demolished structures is currently landfilling. This incurs disposal fees (tipping fees), contributing a positive cost to the CEoL term, and results in a residual value (VR) of zero.

Significant research has been dedicated to developing viable recycling technologies for CFRP, which generally fall into three categories: mechanical grinding, thermal processes and chemical processes [39]. From a purely economic standpoint, however, none of these recycling methods have achieved widespread commercial viability for post-construction waste. The energy and processing costs to produce high-quality recycled carbon fibre (rCF) are often still comparable to or higher than the cost of producing industrial-grade carbon fibre [40]. The lack of a stable, high-volume market for rCF further hinders the development of a profitable recycling industry akin to that of steel.

Therefore, in a current LCCA, the EoL phase for a CFRP system represents a modest net cost, whereas for a steel system, it can represent a net credit. While this currently disadvantages CFRP in a whole-life comparison, it is a field of intense research and development. The global push for a circular economy is accelerating innovation in recycling technologies and new, more easily recyclable matrix chemistries (e.g., thermoplastics or vitrimers), suggesting that this economic gap is likely to narrow significantly in the future.

To address these end-of-life limitations, emerging sustainability solutions are rapidly advancing. Modern pyrolysis recycling techniques can now recover carbon fibres that retain 90–95% of their original tensile strength, consuming only 5–10% of the energy required for virgin fibre production [41]. Furthermore, the transition towards biomaterials derived from renewable precursors, alongside dynamic polymer matrices like vitrimers, provides a viable pathway for closed-loop recycling. These innovations are poised to transform CFRP from a disposal liability into a circular economy asset [42].

### 3.5. LCCA Comparison of CFRP and Traditional Steel Sheet

**Figure 2:**
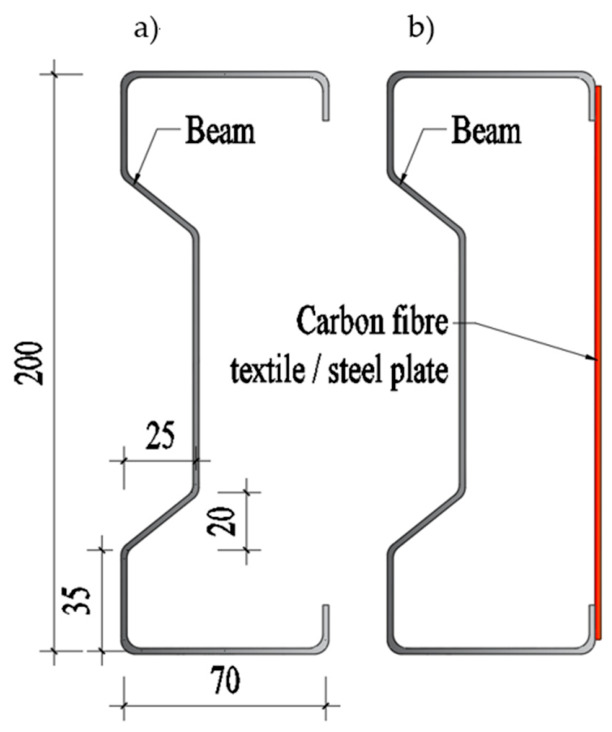
The sigma profile (**a**) bare beam (**b**) reinforced beam-red line.

As part of the case study, an LCCA was conducted for an innovative reinforcement method used on Σ200 × 70 × 2 beams. The proposed reinforcement method involves connecting the upper and lower flanges of the beam by bonding unidirectional carbon fibre textile or steel sheet (thickness of 2 mm), which creates a closed cross-section equal to the width of the reinforcement. Using a carbon fibre textile as a restraint, by bonding the textile to sectionally close the open profile, the reinforcement acts as a tension element that physically prevents the cross-section from “opening,” thereby neutralizing the distortional failure mode and mitigating warping. The analyzed reinforcement method is presented in Figure 2.

To ensure the scientific validity of the LCCA, it is imperative to establish strict structural equivalence between the compared alternatives. The economic assessment must be normalized by functional performance, utilizing a “cost-per-unit capacity” or strength-to-cost ratio approach [33]. While the 2.0 mm thick steel sheet provides the necessary tension constraint to neutralize the distortional failure mode, standard structural steel typically exhibits a yield strength of 235 to 500 MPa. In contrast, the unidirectional CFRP textile operates with an ultimate tensile strength ranging from 2500 to 3500 MPa [43]. Consequently, the identical structural stiffness and strength enhancements required to prevent the cross-section from opening are achieved using a significantly smaller cross-sectional area of the CFRP textile. By normalizing the initial investment against the actual structural capacity delivered rather than raw material volume, both alternatives provide equivalent functional performance, ensuring the economic comparison is structurally sound.

**Figure 3:**
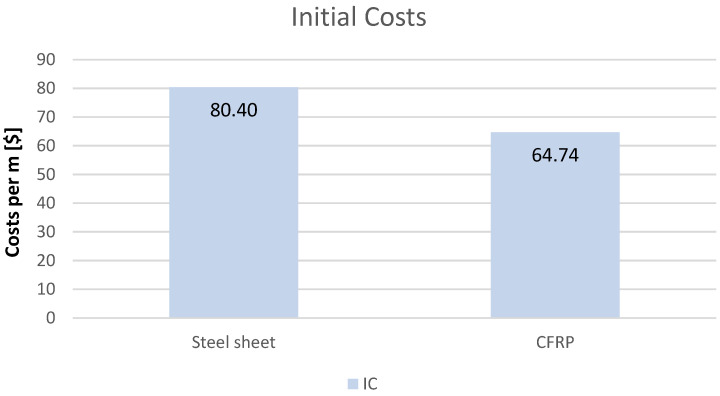
LCCA (IC) comparison.

In economic analyses of structural reinforcement technologies, it is primarily the initial costs that can be reliably quantified. These include the price of materials, labour, and equipment, as well as indirect costs, which are usually estimated on the basis of fixed percentage rates applied to direct costs. The graph shows a graphical representation of the costs for steel and CFRP, a value that can be expressed numerically (Figure 3).

When calculating the cost of labour, materials and equipment, the previously cited data on percentage differences for both solutions and the Sekocenbud price list for the second quarter of 2025 (assuming an exchange rate of PLN 3.61 to the dollar) and indirect costs (during construction process) at 30% are taken into account [44]. In the case of CFRP (carbon fibre-reinforced polymer) systems, the initial costs are usually up to five times higher than for traditional steel solutions when analyzing the cost of the material alone. However, once the lower number of workers required to do the job and the equipment required for steel construction is taken into account, this cost reverses in favour of CFRP, and savings can reach 33% at a fairly early stage.

In this paper, a PERT (Programme Evaluation and Review Technique) analysis was also carried out in order to detail and justify the scores presented in the comparative table. The PERT method involves the use of three estimates of the analyzed parameter (minimum, most probable and maximum), on the basis of which the expected value and measure of variability are calculated, allowing for the uncertainty of the input data (Formula (2)) and in order to determine the standard deviation (Formula (3)). The analysis was used to compare steel and CFRP materials, taking into account criteria such as cost, recycling cost, recovery rate, service life and annual maintenance cost. In Table 2, the values for each parameter are summarized and their expected values and deviations according to the PERT method are calculated, which enabled a more objective assessment and comparison of the analyzed materials [45].

(2) $$PERT=\frac{\left(O+4L+P\right)}{6}$$

(3) $$Deviation=\frac{\left(P-O\right)}{6}$$

O—Optimistic value;
L—Most likely value;
P—Pessimistic value.

The PERT analysis compared steel and CFRP materials, taking into account parameters such as purchase cost, recycling cost, recovery rate, dismantling cost, service life and annual maintenance costs. Although the cost of CFRP material itself is significantly higher than that of steel, when taking into account the total expenditure on labour, materials and equipment, the final use of CFRP in the investment proves to be more cost-effective. The other parameters show typical differences: steel has a higher recovery rate and lower recycling costs, while CFRP has lower annual maintenance costs and comparable durability, which, combined with lower operating costs, can offset the higher initial cost. The table shows the minimum, most likely and maximum values for each parameter, and the expected value and standard deviation have been calculated using the PERT method.

It is much more difficult to clearly express the subsequent costs resulting from LCCA in figures. For this purpose, a five-point scale was adopted to assess each stage and is described in Table 3. It will not provide a clear answer to the cost differences, but it will allow us to present the estimated difference between the traditional solution and the use of CFRP.

1—Estimate that translates into low cost; 5—Estimate that translates into high cost.

**Figure 4:**
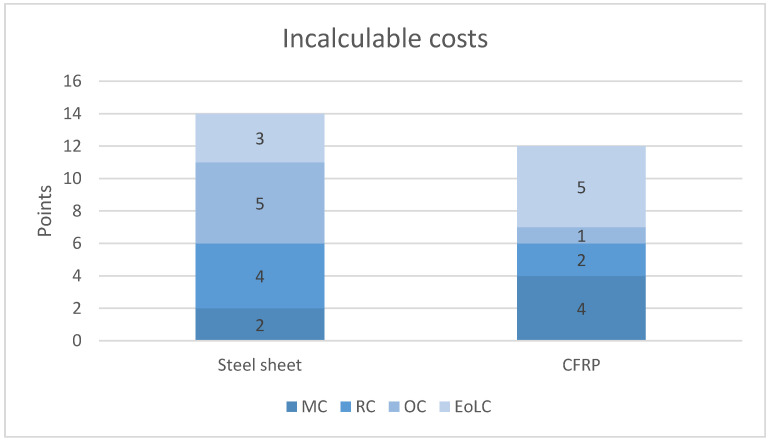
LCCA (MC, OC, RC, EoLC) comparison.

After collecting points (Figure 4) for maintenance costs, repair and rehabilitation costs, and operational costs, it is clear that CFRP outperforms steel in terms of operating costs. Although maintenance costs (MCs) are slightly higher for CFRP, lower repair costs (RCs) and significantly shorter downtime (OC) result in lower total life-cycle costs than for steel by almost 25%. Consequently, from an LCCA perspective, CFRP technology is more cost-effective in terms of facility operation.

## 4. Market Dynamics and Future Trends

The overall economic viability of CFRP strengthening is shaped by a dynamic interplay of current market forces and forward-looking technological trends. An analysis of these factors is crucial for understanding the technology’s long-term competitive position. Table 4 summarizes the key market and institutional factors that currently influence the cost and adoption of CFRP.

While current market forces are making CFRP more cost-effective, the next wave of economic and performance gains will be driven by technological innovation. Table 5 outlines the most significant advanced developments and future trends that will shape the future of fabric-based strengthening.

In conclusion, the techno-economic profile of CFRP strengthening is continuously improving. The baseline costs are decreasing due to maturing market forces [55,56], while ongoing technological innovation is creating systems that are not only more effective but also smarter, more sustainable, and economically viable for an expanding range of applications.

## 5. Conclusions and Research Outlook

This techno-economic review has systematically analyzed the multifaceted economic case for utilizing carbon fibre fabrics and related composite systems in structural strengthening. The analysis demonstrates that a paradigm shift from traditional, initial-cost-based evaluation to a comprehensive, life-cycle framework is essential to fully appreciate the value proposition of these advanced materials. While the upfront investment in CFRP systems is demonstrably high, a holistic assessment consistently reveals their potential to be the most economically prudent long-term solution for a wide range of infrastructure challenges.

### 5.1. Summary of Key Findings

- The primary economic characteristic of CFRP strengthening is its inverted cost structure compared to conventional methods. The high initial material cost is significantly offset by savings in labour, equipment, and, most critically, the massive indirect costs associated with public disruption and facility downtime. This makes CFRP particularly valuable for projects on critical infrastructure where time is a key economic driver.
- The core of CFRP’s economic advantage lies in its long-term performance. Its immunity to corrosion [11] virtually eliminates the substantial future maintenance and repair costs that are inherent to steel-based systems [37]. When evaluated over a typical service life of 50 years or more, the lower Net Present Value (NPV) of these future expenditures often makes CFRP the most cost-effective choice, despite its higher initial cost.
- The economic benefits of CFRP are application-specific. For bridges, the value is primarily in minimizing user delay costs [76]. For seismic retrofitting, it is in the immense value of avoided catastrophic loss, resulting in high benefit–cost ratios [77]. For heritage structures, the value lies in preserving irreplaceable cultural and economic assets through compatible, minimally invasive interventions [67], while for industrial facilities, it is in maximizing revenue through rapid return-to-service [78].
- The economic viability of CFRP is continuously improving. While subject to supply chain volatility [54], the overall cost of CFRP systems is on a downward trend, driven by economy of scale in manufacturing and the increased proficiency and standardization of application techniques guided by established codes like the ACI 440 [18].

### 5.2. Identification of Knowledge Gaps and Challenges

Despite the clear benefits, several challenges and knowledge gaps remain that temper the universal adoption of CFRP and require further attention:

- The lack of a cost-effective, scaled-up recycling industry for post-construction CFRP waste remains the most significant challenge to its long-term sustainability and a weak point in its life-cycle economic profile [39].
- The poor performance of standard epoxy resins at elevated temperatures remains a concern, often requiring the addition of costly passive fire protection systems, which can negatively impact the economic case for CFRP in certain building applications. The development of TRM/FRCM systems [66] is a direct response to this challenge.
- There is a need for more robust, long-term field data to refine LCCA and CBA models. This includes better quantification of the real-world economic value of the data provided by SHM systems [72] and more case studies tracking performance and costs over multiple decades.

Furthermore, a notable limitation of the current research is its reliance on a deterministic life-cycle cost analysis (LCCA) framework. While the application of the PERT method effectively mitigates uncertainty within the initial input variables, the final economic conclusions regarding the comparative superiority of CFRP remain deterministic. Over extended service lives, life-cycle cost outcomes are inherently sensitive to several highly volatile parameters, most notably the applied discount rate, future maintenance frequencies, assumed service life, and the monetization of indirect operational downtime costs. Because empirical, long-term variance data for this novel CFRP structural application is currently scarce, establishing arbitrary variance bounds for sensitivity analysis may introduce speculative bias. Therefore, this study establishes a stable baseline utilizing expected values. It is imperative that as long-term field data becomes more robust, future research must subject these baseline findings to rigorous sensitivity analyses and probabilistic modelling to fully demonstrate how changes in these critical economic parameters influence the long-term viability of composite strengthening systems.

### 5.3. Future Research Directions

To address these challenges and further enhance the techno-economic position of fabric-based strengthening, future research should focus on the following high-impact areas:

- Prioritize research into energy-efficient recycling processes and the development and commercialization of novel, inherently recyclable matrix systems, such as vitrimers [65], to create a closed-loop life-cycle for composites.
- Expand the capabilities of SHM-integrated systems [71] beyond simple monitoring. Research should explore the integration of additional functionalities, such as self-heating capabilities for de-icing bridge decks or energy-harvesting properties, thereby adding more streams of value to the cost–benefit analysis.
- Accelerate the development and deployment of robotic application systems [79] to reduce labour costs and improve quality. Furthermore, research is needed on integrating SHM data with “digital twins” of strengthened structures to enable predictive maintenance and more accurate service life forecasting.
- Develop more sophisticated LCCA and CBA models that can better quantify the economic value of enhanced resilience, risk reduction [80], and socio-economic co-benefits, providing policymakers with a clearer and more compelling case for investment in advanced rehabilitation technologies.

In conclusion, the economic narrative of carbon fibre fabrics in construction is one of evolving and expanding value. Moving beyond a simplistic comparison of initial costs, a mature techno-economic analysis reveals a technology whose true worth is realized over decades of durable service, reduced societal disruption, and enhanced safety.

However, it must be emphasized that CFRP is not a universal solution. In operational contexts where user delay costs (WZRUC) are negligible, or where strict fire-resistance codes mandate the application of costly passive fire protection, conventional methodologies such as steel plate bonding and reinforced concrete jacketing often remain structurally and economically preferable. Ultimately, the optimal selection of a strengthening system requires a nuanced, asset-specific evaluation, positioning CFRP as a highly effective, yet conditional, instrument for modern infrastructure management.

## Figures and Tables

**Table 1:** Comparative unit material costs for structural strengthening systems (USD/kg).

Strengthening System	Typical Material Cost (% of CFRP Cost)
CFRP textile (wet layup)	100%
Steel plate bonding	~10%
Glass fibre-reinforced polymer	~25%

Source: [10,14,17].

**Table 2:** PERT analysis.

Parameter	Unit	Min (P)	Most Likely (L)	Max (O)	PERT	Deviation	Source
Steel							
Price	EUR/t	600	900	1200	900.00	100.00	[46]
Recycling cost	EUR/t	30	50	80	51.67	8.33	[46]
Recovery rate	%	90	95	98	94.67	1.33	[47]
Dismantling cost	EUR/t	30	50	80	51.67	8.33	[48]
Service life	years	50	75	100	75.00	8.33	[30]
Annual maintenance cost	% initial cost/year	0.5	1	2	1.08	0.25	[49]
CFRP							
Price	EUR/t	20,000	30,000	40,000	30,000.00	3333.33	[50]
Recycling cost	EUR/t	5000	10,000	20,000	10,833.33	2500.00	[38]
Recovery rate	%	40	60	90	61.67	8.33	[38]
Dismantling cost	EUR/t	300	1000	1500	966.67	200.00	[51]
Service life	years	50	75	100	75.00	8.33	[52]
Annual maintenance cost	% initial cost/year	0.2	0.5	1	0.53	0.13	[52]

**Table 3:** Cost difference estimation.

Steel Sheets	CFRP
Maintenance Costs	
The maintenance costs of steel structures are relatively low as the technology is well known and widely used. Visual inspections, corrosion measurements, and technical condition assessments can be carried out by personnel with standard qualifications, using commonly available testing methods. In addition, there is extensive operational experience and clearly defined maintenance procedures, which reduce cost risks. (2/5)	In the case of CFRP, maintenance costs are more difficult to estimate and potentially higher due to the need for specialized diagnostic methods (e.g., non-destructive testing to detect delamination or matrix degradation). Higher staff qualifications and less operational experience are required compared to steel, which justifies a higher score. (3/5)
Repair and Rehabilitation Costs	
Repairs and reinforcements of steel structures are time-consuming and often require welding, dismantling of components, and anti-corrosion protection. This process involves long lead times, a larger scope of work, and significant indirect costs, which translates into a high RC cost assessment. (4/5)	CFRP systems enable rapid repairs and reinforcements using bonding or laminating methods without significant interference with the structure. Shorter lead times, reduced need for heavy equipment, and limited preparatory work result in lower repair and rehabilitation costs throughout the entire life-cycle. (2/5)
Operational Costs	
Work related to the installation, repair, or replacement of steel components often requires the facility to be taken out of service for long periods (weeks), which generates high indirect costs, such as traffic restrictions, economic losses for users, and the costs of organizing replacement transport. For this reason, steel operating costs are considered very high. (5/5)	CFRP technology is characterized by very short installation and repair times (usually days), which significantly reduces operational downtime. Minimal interference with the use of the facility means that operating costs are the lowest among comparable technologies. (1/5)
End-of-Life Cost	
In phases A1–A3, steel has a lower GWP (3.98 kg CO_2_e/kg) compared to CFRP, which is due to mature and optimized production processes. The key advantage of steel becomes apparent in the end-of-life phase, where its high recycling potential allows recycling credits to be obtained, offsetting part of the production emissions. As a result, the total carbon footprint of steel is significantly reduced over its life-cycle. (3/5)	CFRP exhibits a higher GWP in phases A1–A3 (4.5 kg CO_2_e/kg), mainly due to the intensive production of carbon fibres and resins. In the end-of-life phase, limited recycling options mean that there is no significant compensation for the emissions generated during production. As a result, the high carbon footprint of phases A1–A3 remains largely unchanged throughout the life-cycle. (5/5)

**Table 4:** Key market and institutional factors influencing the economic viability of CFRP.

Factor	Key Trend/Outlook	Influence on CFRP Economics
Supply Chain & Material Price	Risk remains, but R&D into low-cost precursors (lignin) [53] and increased production capacity are expected to lead to greater price stability.	High price volatility linked to energy and petrochemical markets (PAN precursor) [16]. Supply can be tightened by demand from other sectors (aerospace, wind) [54].
Economy of Scale & Maturity	Sustained cost reduction. Driven by large-scale manufacturing for automotive/wind industries [54] and a growing, more efficient labour force benefiting from a “learning curve” effect [55].	Historically high costs due to specialized, low-volume production and labour [56].
Competition (Alternative FRPs)	Continued segmentation. CFRP will likely remain the premium choice for critical applications, while the “middle ground” BFRP [57] may gain market share from GFRP.	Market segmentation by performance. CFRP for high-performance applications, GFRP for low-cost needs [19,52].
Codes, Standards & Policy	Strong positive influence. Growing focus on sustainability and life extension in public policy [58] inherently favours strengthening over replacement, further boosting the market for CFRP.	Risk reduction and market creation. Standards like ACI 440 [19,59] reduce engineering risk [60]. Government mandates [61] and infrastructure funding [62] create large-scale demand.

**Table 5:** Advanced developments and future trends in composite strengthening.

Trend/Technology	Description & Rationale	Techno-Economic Impact
Advanced Materials	Development of hybrid composites for pseudo-ductile behaviour [63], nano-engineered matrices for enhanced durability [64], and sustainable, recyclable resins like vitrimers [65].	Optimizes the cost–performance ratio, improves safety and long-term durability, and addresses end-of-life economic and environmental challenges, creating a circular economy.
Inorganic Matrices (TRM/FRCM)	Use of cement-based mortars instead of epoxy resins as the matrix [66]. Motivated by the need for better fire performance, substrate compatibility (especially important for heritage masonry structures) [67], and vapour permeability.	Provides a highly cost-effective solution for fire-critical applications (by eliminating separate fireproofing costs) and heritage conservation, expanding the addressable market.
Automation & Robotics	Integration of robotic systems for surface preparation, automated fabric placement [68], and in-process quality control using sensors [68], addressing labour costs and improving quality [69].	Reduces long-term costs by minimizing labour, increasing speed, and reducing material waste. Improves quality and reliability, offsetting a high initial capital investment.
Structural Health Monitoring (SHM)	Embedding sensors, particularly Fibre Optic Sensors (FOS) [70], directly into the CFRP laminate [71] to enable a shift to condition-based maintenance [72].	The higher initial cost is justified by long-term savings from optimized maintenance, enhanced safety through early-warning systems, and data-driven service life extension.
Sustainability & LCA	Application of life-cycle assessment (LCA) [73], which shows that strengthening is more sustainable than replacement [74], despite the high embodied energy of CFRP. Carbon pricing [75] will make this a direct financial benefit.	Aligns the economic benefits of life extension with sustainability goals. The low environmental impact of strengthening becomes a quantifiable financial advantage, improving the overall business case.

## Data Availability

The original contributions presented in this study are included in the article. Further inquiries can be directed to the corresponding author.
